# Hypothalamic vasotocin and tyrosine hydroxylase levels following maternal care and selection for low mortality in laying hens

**DOI:** 10.1186/1746-6148-10-167

**Published:** 2014-07-31

**Authors:** Susie E Hewlett, Elly C Zeinstra, Frank JCM van Eerdenburg, TB Rodenburg, Peter JS van Kooten, FJ van der Staay, Rebecca E Nordquist

**Affiliations:** 1 Emotion & Cognition Group, Department of Farm Animal Health, Faculty of Veterinary Medicine, Utrecht University, Yalelaan 7, 3584, CL, Utrecht, The Netherlands; 2 Advances in Veterinary Medicine, Department of Farm Animal Health, Utrecht University, Utrecht, the Netherlands; 3 Behavioural Ecology Group, Wageningen University, Wageningen, the Netherlands; 4 Department of Infectious Diseases and Immunology, Division of Immunology, Faculty of Veterinary Medicine, Utrecht University, Utrecht, the Netherlands; 5 Brain Center Rudolf Magnus, University Medical Center Utrecht, Utrecht, the Netherlands

**Keywords:** Hypothalamus, Vasotocin, Vasopressin, Tyrosine hydroxylase, Dopamine, Welfare, Laying hen

## Abstract

**Background:**

Feather pecking and cannibalism are major concerns in poultry farming, both in terms of animal welfare and farm economics. Genetic selection and introduction of (aspects of) maternal care have been suggested as potential interventions to reduce feather pecking in laying hens. Altered brain development has been proposed to reflect welfare states in animals, and can provide more insight into the underlying processes involved in feather pecking. Both vasotocin (the avian homologue of vasopressin) and dopaminergic neural circuitry have roles in control of social behaviors as well as in the stress response, and may be linked to feather pecking. Thus, the hypothalamus of adult laying hens selected for low early mortality (LML), which show low feather pecking, was examined and compared with a control line of adult laying hens selected for production characteristics only (CL). The effect of foster hen rearing on the two genetic lines and their hypothalamic morphology was also investigated.

**Results:**

We demonstrated an increase in the number of neurons positive for the rate-limiting enzyme in dopamine production, tyrosine hydroxylase, in the periventricular area of the hypothalamus in the LML hens compared to CL hens. Hen-reared chicks showed more vasotocin -positive neurons in the medial pre-optic area compared to the hens raised without a hen. No correlations were found between behavior in an open field at 5–6 weeks of age, and the histology of the same hens at adulthood.

**Conclusion:**

The hypothalamic dopaminergic and vasotinergic systems are altered in hens following genetic selection or maternal care, indicating a potential role for these systems in feather pecking.

## Background

Feather pecking and cannibalism are concerning welfare problems in poultry farming, and a major topic of welfare research in laying hens [1-4]. Severe feather pecking compromises the victim’s welfare through physical pain and chronic fear, but also signals that the welfare of the perpetrator is being compromised, leading to the development of this maladaptive coping mechanism [5]. Recently, a line of laying hens was produced following sibling selection for low premature mortality in addition to production characteristics [6]. This line shows low mortality due to feather pecking and cannibalism, and differs from animals selected for production characteristics only for a number of physiological and behavioral measures which may be related to fear or stress, including altered whole-blood serotonin levels, plasma corticosterone (CORT) levels and open field behavior, while leaving cognition intact [3,7-9]. Altered brain development has been proposed as a potential “biomarker” for welfare states in animals [10], and can provide more insight into the underlying processes involved in feather pecking. Based on the above findings the question is raised whether genetic selection, which has produced a reduction in feather pecking, has altered the neuroanatomy of the animals, particularly the brain areas involved in stress responses.

Introduction of (aspects of) maternal care has been proposed as a potential intervention to reduce feather pecking in laying hens. Maternal deprivation has been reported to affect the neuro-endocrine response to stress [11]. Rat pups raised by low care giving mothers show increased CORT responses following an acute restraint test [12] and different cFOS activity patterns in the paraventricular nucleus (PVN) of the hypothalamus after a shock probe stressor test [13] compared to pups raised by high care giving mothers. Moreover, an increase in the hypothalamic-pituitary-adrenal (HPA) axis response correlates with maternally deprived animals’ fearful reactions to novel situations [14]. In young chicks, vocalizations of a mother hen appear to positively affect learning and memory [15], and a maternal odorant lowers stress response in chicks when faced with isolation and novelty [16]. Maternal care in chicks also promotes exploratory behaviors [17]. Both fearfulness [18] and knowledge about potential threats is communicated from (surrogate) mother hens to chicks [19]. Our previous studies also demonstrated that genetic selection against early mortality produced alterations in levels of tyrosine hydroxylase (TH), the rate-limiting enzyme in synthesis of catecholamines including dopamine (DA), in the nidocaudolateral pallium [20], decreased levels of noradrenaline (NA) and the DA metabolite 3,4-dihydroxyphenylacetic acid (DOPAC) and a trend to decreased DA and its metabolite homovanillic acid (HVA) in the arcopallium, and increased DA turnover in the hippocampus [21]. Furthermore, providing maternal care caused alterations in differences in cell size between the two hemispheres in the hippocampus [20].

The HPA axis is the major brain circuit involved in stress responses in many different species, including avians [22]. Male broiler hens that experienced a social stressor had high cFOS immunoreactivity in the PVN, implicating the involvement of the HPA axis in the stress response of poultry [23]. Furthermore, co-administration of corticotropin releasing hormone (CRH) and arginine vasotocin (AVT; a vasopressin orthologue found in birds, reptiles and fish) in the periphery produced a strong CORT release [24], indicating that AVT is a fundamental part of the HPA axis circuitry, as vasopressin is in mammals [25]. AVT (as with vasopressin) has also been strongly implicated in social behaviors, possibly through its interactions with mesotocin, the oxytocin avian homologue [26,27].

Catecholamines, such as DA, along with the glucocorticoids are released in stressful situations and act on the hippocampus and amgydala, affecting emotional states [14]. DA projected from the ventral tegmental area to the PVN stimulates the HPA axis and increases corticotropin-releasing factor (CRF) release in response to a stressor [28]. Belda and colleagues [29] further demonstrated DA stimulation of the HPA axis by blocking DA signaling, resulting in a reduced level of adrenocorticotropic hormone (ACTH) and cortisol (CORT) release in rats recently exposed to a chronic stressor.

These previous results, and the necessity to further explore the potential of characterization of brain areas for use in animal welfare research, led to the question of whether stress-related brain areas could be altered by genetic selection for low early mortality and/or maternal care in laying hens. DA has also been implicated with a role in the expression of feather pecking behavior [30] and, as mentioned above, TH is altered in laying hens from a low mortality selection line [20]. Both AVP and DA neural circuitry have roles in control of social behaviors as well as in the stress response [31,32], which may link to maternal care.

The present study investigated whether genetic selection against early mortality, or early life exposure to a mother hen, are associated with alterations in TH or AVT in subareas of the adult laying hen hypothalamus, and whether this correlates with behavioral measures taken in an open field in early life.

## Results

Results of TH and vasotocin neuron counts in the hypothalamus are summarized in Table 1. Areas and nuclei that were affected by genetic line or rearing condition will be discussed further.

**Table 1:** Average number of immunopositive neurons in each brain area per treatment group and results of statistical analyses

**Brain area**	**Neuron type**	**CL NO**	**CL HEN**	**LML NO**	**LML HEN**	**Main effect: Hen rearing**		**Main effect: Genetic line**		**Interaction: Hen-rearing by Genetic line**	
		**Number of immunopositive neurons**				**F**	**p**	**F**	**p**	**F**	**p**
PaPC	AVT*	108.0 ± 12.7	148.6 ± 29.5	140.1 ± 16.7	123.7 ± 38.4	0.00	1.00	0.05	0.82	2.39	0.14
	TH	NA	NA	NA	NA	NA	NA	NA	NA	NA	NA
PaMC	AVT	65.0 ± 12.5	104.9 ± 26.9	68.1 ± 13.7	61.3 ± 10.1	0.02	0.89	0.76	0.39	2.02	0.17
	TH	NA	NA	NA	NA	NA	NA	NA	NA	NA	NA
whole PVN	TH	39.3 ± 8.1	49.1 ± 3.1	44.9 ± 3.4	39.8 ± 7.7	0.00	0.95	0.50	0.49	1.04	0.32
MPO	AVT	30.4 ± 8.2	35.9 ± 10.9	24.0 ± 7.8	29.4 ± 7.9	0.18	0.68	0.56	0.46	0.18	0.68
	TH	NA	NA	NA	NA	NA	NA	NA	NA	NA	NA
MPA	AVT	6.3 ± 2.8	28.4 ± 12.7	5.9 ± 3.8	37.7 ± 9.9	*6.70*	*0.02*	0.45	0.51	1.07	0.31
	TH	NA	NA	NA	NA	NA	NA	NA	NA	NA	NA
AMPO	AVT	18.4 ± 5.3	32.4 ± 5.4	31.7 ± 6.8	31.4 ± 6.4	1.63	0.21	1.08	0.31	1.88	0.18
	TH	NA	NA	NA	NA	NA	NA	NA	NA	NA	NA
LPO	AVT	60.6 ± 10.1	72.1 ± 10.5	56.1 ± 13.0	97.7 ± 19.5	*4.25*	*0.05*	0.52	0.48	0.59	0.45
	TH	NA	NA	NA	NA	NA	NA	NA	NA	NA	NA
whole POA	TH	26.7 ± 16.0	5.9 ± 5.9	36.0 ± 13.9	15.3 ± 11.5	2.58	0.12	1.04	0.32	0.00	0.97
SON	AVT	177.1 ± 52.8	172.9 ± 18.6	173.9 ± 40.7	168.1 ± 41.8	0.00	0.97	0.21	0.65	0.17	0.68
	TH	NA	NA	NA	NA	NA	NA	NA	NA	NA	NA
AH	AVT	16.1 ± 6.5	37.7 ± 9.9	15.3 ± 5.0	17.1 ± 5.2	2.15	0.16	1.44	0.24	1.88	0.18
	TH	16.7 ± 5.2	16.1 ± 2.6	13.9 ± 1.1	11.3 ± 4.3	0.08	0.78	1.35	0.26	1.26	0.27
LH	TH	20.9 ± 3.9	30.1 ± 6.8	30.1 ± 5.6	30.5 ± 6.6	0.30	0.59	0.60	0.45	0.29	0.60
VMH	AVT	NA	NA	NA	NA	NA	NA	NA	NA	NA	NA
	TH	2.1 ± 0.6	3.9 ± 1.0	5.9 ± 2.3	2.5 ± 0.6	0.02	0.88	0.15	0.71	1.27	0.27
PH	AVT	NA	NA	NA	NA	NA	NA	NA	NA	NA	NA
	TH	25.6 ± 5.2	22.9 ± 7.0	28.6 ± 5.3	23.0 ± 4.8	0.44	0.51	0.00	0.95	0.00	0.94
DM	AVT	NA	NA	NA	NA	NA	NA	NA	NA	NA	NA
	TH	2.0 ± 1.2	3.6 ± 1.7	0.7 ± 0.5	3.7 ± 1.5	*5.65*	*0.03*	0.28	0.60	0.44	0.52
HSOD	TH	6.7 ± 3.6	8.4 ± 1.9	6.4 ± 1.2	10.8 ± 3.6	1.48	0.24	0.30	0.59	0.23	0.64
Pe	AVT	NA	NA	NA	NA	NA	NA	NA	NA	NA	NA
	TH	15.0 ± 3.2	13.9 ± 3.4	26.4 ± 3.2	24.5 ± 6.9	0.41	0.53	*7.09*	*0.01*	0.14	0.71
ML/MM	AVT	NA	NA	NA	NA	NA	NA	NA	NA	NA	NA
	TH	1.3 ± 1.3	3.7 ± 3.2	4.4 ± 2.8	5.3 ± 4.8	0.31	0.58	0.41	0.53	0.63	0.44
RM	AVT	NA	NA	NA	NA	NA	NA	NA	NA	NA	NA
	TH	12.4 ± 3.0	24.4 ± 6.7	24.7 ± 4.8	19.5 ± 2.1	0.78	0.39	1.71	0.20	2.26	0.15
maVTA	AVT	NA	NA	NA	NA	NA	NA	NA	NA	NA	NA
	TH	0.0 ± 0.0	0.0 ± 0.0	27.7 ± 18.2	10.2 ± 10.2	0.32	0.58	3.66	0.07	0.35	0.56

Total count for each brain was divided by the number of sections containing a brain area. Results presented as average ± SEM; analysis was performed on ranks with a Friedman's Two-way Nonparametric ANOVA. * Is the average result per treatment group but not by the dividing number of sections containing neurons in the PaPC.

*Abbreviations:PaPC* parvocellular area of the paraventricular nucleus, *PaMC* magnocellular area of the paraventricular nucleus, whole *PVN* All areas of the paraventricular nucleus of the hypothalamus, *MPO* medial pre-optic nucleus, *MPA* Medial pre-optic area, *AMPO* anteromedial pre-optic nucleus, *LPO* Lateral pre-optic nucleus, whole *POA* all preoptic nuclei, *SON* supraoptic nucleus, *AH* anterior hypothalamic nucleus, *LH* lateral hypothalamic nucleus, *VMH* ventromedial hypothalamic nucleus, *PH* posterior hypothalamic nucleus, *DM* dorsomedial hypothalamic nucleus, *HSOD* hypothalamic nucleus of the supraoptic decussation, *Pe* periventricular area, *ML/MM* medial and mediolateral part of the mammillary nucleus, *RM* retromammillary area, *BNST* bed nucleus of the stria terminalis, *maVTA* mammillary part of the ventral tegmental area. *NA* not applicable as neuron type not found in that area. Significant effects (p<0.05) are indicated in italics.

### Tyrosine hydroxylase (TH)

**Figure 1:**
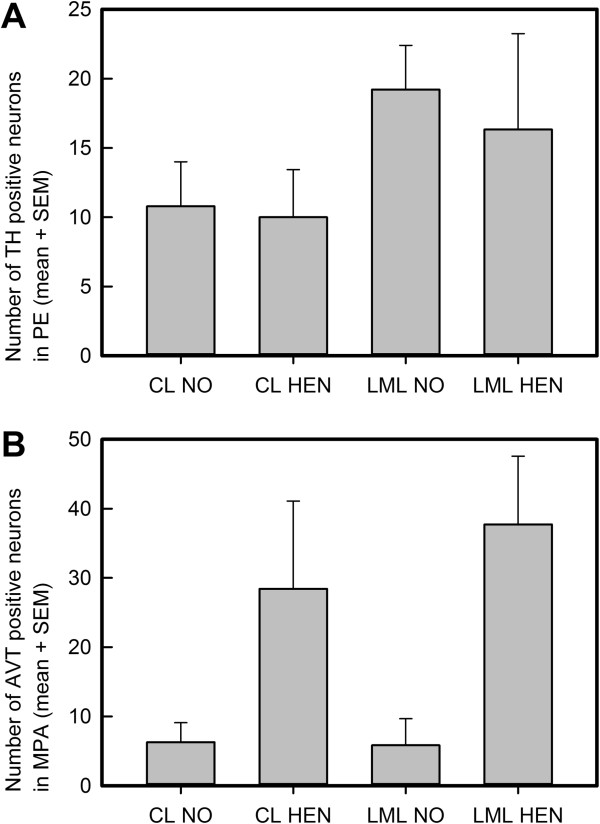
**Graphic representation of the average number of TH- positive neurons in the Pe and AVT- positive neurons in the MPA. A)** The average total number of tyrosine hydroxylase neuronal cells per containing sections in the periventricular area (Pe) of the hypothalamus (significant main effect of genetic line, see Table 1), and **B)** the average total number of vasotocin neuronal cells per containing sections in the medial pre-optic area (MPA; significant main effect of rearing, see Table 1). Results presented as average ± SEM; analysis was performed on ranks with a Friedman's Two-way Nonparametric ANOVA. CL NO = control genetic line raised without a hen, CL HEN = control genetic line raised with a hen, LML NO = low mortality genetic line raised without a hen, LML HEN = low mortality genetic line raised with a hen.

**Figure 2:**
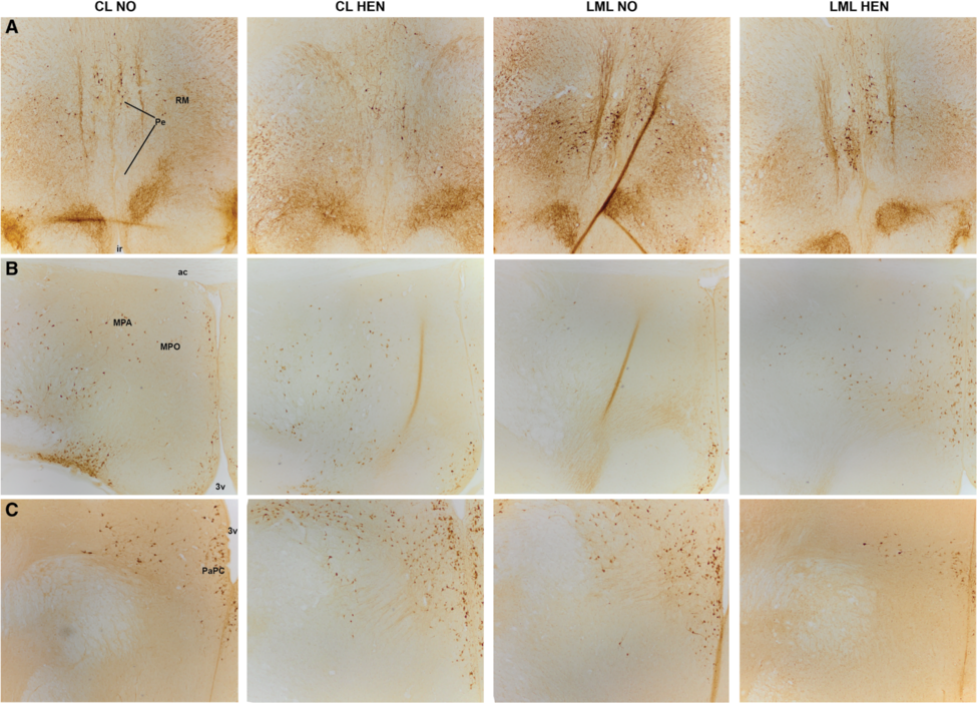
**Photomicrographs of TH and AVT immunohistochemical staining in the hypothalamus. A)** Photomicrographs of tyrosine hydroxylase neuronal cells in the periventricular area (Pe) of the hypothalamus, based on figure 25 of the stereotaxic atlas of the chick brain (Puelles et al. [48]), **B)** Photomicrographs of vasotocin neuronal cells in the medial pre-optic area (MPA), based on figure 14 of the stereotaxic atlas of the chick brain (Puelles et al. [48]) and **C)** Photomicrographs of vasotocin neuronal cells in the parvocellular part of the paraventricular nucleus (PaPC), based on figure 16 of the stereotaxic atlas of the chick brain (Puelles et al. [48]). Left to Right: CL NO = control genetic line raised without a hen, CL HEN = control genetic line raised with a hen, LML NO = low mortality genetic line raised without a hen, LML HEN = low mortality genetic line raised with a hen. All images were taken at 10x magnification. Abbreviations: 3v = third ventricle, ac = anterior commissure, ir = infundibular recess, MPO = medial pre-optic nucleus, MPA = Medial pre-optic area, PaPC = parvocellular area of the paraventricular nucleus, Pe = periventricular area.

More TH containing cells were counted in the periventricular area of the hypothalamus (Pe) in the LML compared to the CL hens (F_1, 23_ = 7.09, p = 0.01; visualized in Figures 1A and 2). There was no effect of rearing condition on the number of TH immunoreactive cells in the Pe (F_1, 23_ = 0.41, p = 0.53). In the DM, more TH immunopositive neurons were found in hen-reared chickens compared to those reared without a hen (F_1, 23_ = 5.65, p = 0.03), although the average number of neurons counted was low (overall average of 2.4 ± 0.65 neurons counted per section containing the DM). No effect of genetic line was seen in the DM (F_1, 23_ = 0.52, p = 0.48).

Selection line and rearing condition did not affect the number of TH-ir cells in any other areas of the hypothalamus.

### Vasotocin

Rearing condition affected the number of AVT-ir cells in the medial pre-optic area of the hypothalamus (MPA), as chickens raised with a hen and receiving maternal care in early life had more AVT-ir cells in the MPA compared to the hens raised without a mother hen (F_1,24_ = 6.70, p = 0.02, visualized in Figures 1B and 2).

The chickens raised with a mother hen also tended to have more AVT-ir cells in the lateral pre-optic area of the hypothalamus (LPO) compared to the chickens raised without a hen (F_1,24_ = 4.25, p = 0.05).

### Correlations with behavioral data

Correlations were observed between several behavioral measures with other behavioral measures, such as between latency to stand and latency to walk, between latency to stand and number of steps, and between latency to stand and number of distress calls. Correlations were also found between neuron counts with other neuron counts, for instance between TH positive neurons in VMH and TH positive neurons in DM. However, no significant correlations were found between any of the variables from the previously conducted open field test Rodenburg et al. [8,9] and the hypothalamic areas showing significant groups differences in the present study (see Table 2).

**Table 2:** Spearman Rank Correlations between results of open field (OF) at 5–6 weeks of age and number of hypothalamic tyrosine hydroxylase (TH)- or vasotocin (AVT) positive neurons

	**Lstand**	**Lwalk**	**Nstep**	**Ndiss**	**AVT-MPA**	**AVT-PaPC**	**AVT-LPO**	**TH-VMH**	**TH-DM**	**TH-RM**	**TH-PE**
Lstand	1.000	*0.753*	*-0.635*	*-0.580*	-0.108	0.225	0.137	0.031	-0.212	0.060	-0.262
latency to stand in OF		*<.0001*	*0.000*	*0.002*	0.593	0.260	0.495	0.882	0.297	0.770	0.196
	27	*27*	*27*	*27*	27	27	27	26	26	26	26
Lwalk	*0.753*	1.000	*-0.780*	*-0.432*	-0.113	0.220	0.116	0.005	0.012	-0.001	-0.266
latency to walk in OF	*<.0001*		*<.0001*	*0.024*	0.576	0.271	0.565	0.980	0.954	0.997	0.189
	*27*	27	*27*	*27*	27	27	27	26	26	26	26
Nstep	*0.635*	*-0.780*	1.000	*0.546*	0.220	-0.026	-0.065	-0.034	0.051	0.098	0.285
Number of steps in OF	*0.000*	*<.0001*		*0.003*	0.270	0.898	0.748	0.869	0.804	0.633	0.158
	*27*	*27*	27	*27*	27	27	27	26	26	26	26
Ndiss	*0.580*	*-0.432*	*0.546*	1.000	0.110	-0.106	-0.269	0.327	0.241	0.188	0.276
Number of distress calls in OF	*0.002*	*0.024*	*0.003*		0.586	0.599	0.175	0.103	0.237	0.358	0.172
	*27*	*27*	*27*	27	27	27	27	26	26	26	26
AVT-MPA	-0.108	-0.113	0.220	0.110	1.000	0.256	0.303	0.131	-0.134	0.146	0.338
AVT positive neurons in MPA	0.593	0.576	0.270	0.586		0.188	0.117	0.514	0.505	0.467	0.085
	27	27	27	27	28	28	28	27	27	27	27
AVT-PaPC	0.225	0.220	-0.026	-0.106	0.256	1.000	0.217	0.166	-0.008	0.280	*0.545*
AVT positive neurons in PaPC	0.260	0.271	0.898	0.599	0.188		0.268	0.408	0.968	0.157	*0.003*
	27	27	27	27	28	28	28	27	27	27	*27*
AVT-LPO	0.137	0.116	-0.065	-0.269	0.303	0.217	1.000	-0.007	-0.017	0.141	0.013
AVT positive neurons in LPO	0.495	0.565	0.748	0.175	0.117	0.268		0.971	0.932	0.484	0.950
	27	27	27	27	28	28	28	27	27	27	27
TH-VMH	0.031	0.005	-0.034	0.327	0.131	0.166	-0.007	1.000	*-0.392*	*0.744*	*0.514*
TH positive neurons in VMH	0.882	0.980	0.869	0.103	0.514	0.408	0.971		*0.043*	*<.0001*	*0.006*
	26	26	26	26	27	27	27	27	*27*	*27*	*27*
TH-DM	-0.212	0.012	0.051	0.241	-0.134	-0.008	-0.017	*-0.392*	1.000	-0.342	-0.287
TH positive neurons in DM	0.297	0.954	0.804	0.237	0.505	0.968	0.932	*0.043*		0.080	0.147
	26	26	26	26	27	27	27	*27*	27	27	27
TH-RM	0.060	-0.001	0.098	0.188	0.146	0.280	0.141	*0.744*	-0.342	1.000	*0.422*
TH positive neurons in RM	0.770	0.997	0.633	0.358	0.467	0.157	0.484	*<.0001*	0.080		*0.028*
	26	26	26	26	27	27	27	*27*	27	27	*27*
TH-PE	-0.262	-0.266	0.285	0.276	0.338	*0.545*	0.013	*0.514*	-0.287	*0.4221*	1.000
TH positive neurons in Pe	0.196	0.189	0.158	0.172	0.085	*0.003*	0.950	*0.006*	0.147	*0.028*	
	26	26	26	26	27	*27*	27	*27*	27	*27*	27

For all cells in table, correlations are listed as Rho value, P value, and N. See Table 1 for abbreviations of hypothalamic areas. Significant correlations (p<0.05) are indicated in italics.

## Discussion

The hypothalamic brain areas and nuclei of adult laying hens selected for low mortality (LML line) were examined and compared with adult laying hens not selected for specific behavioral traits (CL line). The effect of mother hen rearing on the two genetic lines and their hypothalamic morphology was also investigated. Both TH- and AVT- positive neurons were found in hypothalamic areas and nuclei consistent with previous reports in both avians and mammals (TH: [33]; AVT: [34]). We demonstrated an increase in the number of TH-positive neurons in the Pe of the hypothalamus in the LML hens compared to CL hens. Hen-reared chicks showed more AVT-positive neurons in the MPA compared to the hens raised without a hen. An interaction of genetic selection and hen rearing was observed in the parvocellular part of the PaPC, though none of the groups differed significantly in post-hoc testing. No correlations were found between behavior in an open field at 5–6 weeks of age, and the histology of the same hens at adulthood.

### Genetic selection against feather pecking linked to more TH-positive neurons in periventricular area

TH is a well-established indicator of catecholamine neuron types. The distribution of TH-ir cells in this study matches with previous reports of dopamine neuron distribution, strengthening the reliability of using this rate-limiting enzyme [35]. Moreover, previous studies have shown that while noradrenergic fibers and receptors are present in the hypothalamus, when enzymes used to define noradrenergic neurons (another catecholamine), β-hydroxylase or phenylethanolamine-N-methyltransferase (PNMT) are examined, perikarya of these neuron types are not found in the hypothalamus of mammals [35,36] or are sparsely found, but only in neurons that do not express TH [37]. These observations indicate a high probability that the neurons stained by TH are indeed dopaminergic neurons.

The LML hens had more TH immunopositive neurons in the Pe of the hypothalamus compared to the CL line hens originating from the same commercial breed (White Leghorn). There is evidence for involvement of Pe DA (DA_A14_) neurons in stress responses. In macaques it was found that the DA_A14_ neurons of the Pe area are co-localized with and activated by CRF neurons [38]. Secondly, the Pe area includes part of the magnocellular division of the PVN (PaMC) [39]. The PaMC was recently demonstrated as an integral part of the stress response by releasing peptides peripherally that feedback and activate the HPA axis [24]. Moreover, Moons and colleagues [33] described the dopaminergic cells of the Pe zone as in contact with the PVN region. Dopamine is postulated to play a role in feather pecking, as chickens administered DA receptor antagonists showed reduced feather pecking behavior [30]. In line with these reports, the fourth generation of the same LML hens was shown to have lower corticosterone levels than the control line, and expressed less fear in behavioral testing [3,8,9].

Given the connection between feather pecking and a heightened stress response, it is possible that the present data reflect a role for DA neurons of the Pe in the stress response and feather pecking behavior. If differences in Pe brain neuroanatomy are involved in an altered stress response, as hypothesized by previous researchers [33,38], and are linked to selection against feather pecking as suggested by the present study, Pe DA neuron number may be a useful welfare biomarker in poultry farming. If so, an increase in DA neuron number in the Pe may reflect a propensity to decreased welfare.

More TH-ir cells were found in the Pe of hens from the LML line than in those from the CL line. Our previous studies showed reductions in TH in the nidocaudolateral pallium [20], decreased levels of noradrenaline (NA, for which TH is also a precursor) and the DA metabolite 3,4-dihydroxyphenylacetic acid (DOPAC) and a trend to decreased DA and its metabolite homovanillic acid (HVA) in the arcopallium [21]. More cells in an area increase the likelihood of increased peptide synthesis and release to efferent brain areas, though more immunopositive cells does not strictly mean more neurotransmitter release. However, the increase in TH positive neurons found in the Pe in the present study may be more related to the reproductive role for this area, than feather pecking per se. Hens from LML start egg production later and gain weight more slowly than hens from the control line [40], indicating a change in rate of reproductive maturity as a result of genetic selection. In mammals, the Pe is known to be rich in gonadal steroid receptors [41] and is well innervated by growth hormone-releasing hormone from the arcuate nucleus and ventromedial hypothalamus. Furthermore, the TH cells of the periventricular pre-optic area are colocalized with GnRH and thought to be involved in reproductive physiology [42]. In avians, hypothalamic TH has been correlated with nesting and brooding behavior, with high numbers of TH-positive neurons in nesting (thus non-laying) Thai hens [43,44].

### Maternal care and the medial pre-optic area

AVT is a key neurotransmitter of brain systems controlling social behavior (reviewed: [31]), making it an interesting neuron type to investigate the control of bird-to-bird feather pecking behavior and maternal care in chickens. In the present study, the chickens that had been reared by a foster hen had markedly more AVT neurons in the MPA of the hypothalamus as adults compared to the hens that never experienced maternal care. Considering the 45 week gap between last being housed with a mother hen and euthanasia, this represents a long-lasting effect. Our previous studies in this same group of hens also demonstrated that maternal care alters the difference in cell size between the left and right hippocampus when measured in adult hens [20], supporting the long-lasting effects of maternal care on brain development in laying hens.

The MPA is heavily involved in expression of maternal behavior, and the presence of pups increases the activity of the MPA in female rats [45]. Maternal behavior in commercial laying hen breeds has disappeared- indeed, broodiness is considered a problem [46]. The data from the present study show that experience of maternal care increases cell number in this area, suggesting that although they do not show maternal care, chicks are still receptive to the effects of maternal care. Maternal care also had clear effects on the behavior of the birds in the present study, as they were less fearful during early life compared with non-brooded birds and showed less cannibalistic toe pecking as adults [8]. Testing aspects of maternal care, such as broodiness, of hen reared and non-hen reared chickens would be interesting to see if receiving maternal care positively affects future maternal care giving, as has been established in rodents [47], although this would have to be tested in non-commercial lines, since commercial lines show such low levels of maternal care. If so, it would indicate the importance of maternal care experience on brain development in chickens and have welfare implications for current chick raising methods.

### Correlations with behavioral studies

We did not find any significant correlations between behavior as measured in an Open Field at 5–6 weeks of age, and histological analysis of TH and AVT in the hypothalamus. Although both measures are related to the HPA axis, the lack of a one-to-one relationship between behavior and histology is not surprising, especially given the amount of time which passed between the (relatively short) behavioral study and the time that the animals were sacrificed for histology. Future studies examining correlations with histology should include more animals and/or behavioral measures closer in time to the point reflected in histology.

## Conclusions

In summary, the number of TH-ir neurons in the hypothalamic regions involved in the stress response was altered in animals selected for lower mortality by severe feather pecking. The differences may contribute to behavioral and physiological fear and stress differences observed between the LML and the CL animals.

In addition, differences of AVT neuron number in hypothalamic regions involved in social behavior, specifically maternal behavior, coincided with whether the hens had been raised by a mother hen. This long term and profound anatomical difference is impressive and warrants further investigation into whether the development of feather pecking can be reduced or avoided by rearing production chicks with mother hens or providing aspects of maternal care, such as shelter, warmth, or darkness.

## Methods

The study was reviewed and approved by the local ethics committee of Wageningen University, the Netherlands, and was conducted in accordance with the recommendations of the EU directive 86/609/EEC. All effort was taken to minimize the number of animals used and their suffering.

### Animals

A total of 28 chickens from two selected lines, both originating from the pure bred White Leghorn layer line at ISA B.V. Breeding Division of Hendrix Genetics were used. One line was selected for low premature mortality (LML) according to a sib-selection approach [6]. The second generation of this LML was used. The other genetic line was selected using individual performance only and is referred to as the control line (CL) [8,9]. The animals were raised either with or without a foster hen, producing four treatment groups: LML raised with a hen (LML HEN), LML raised without a hen (LML NO), CL raised with a hen (CL HEN), and CL raised without a hen (CL NO). For details of the rearing and fostering procedures, see Rodenburg et al. [8,9]. At week 52, all hens (n = 7 per group) were humanely euthanized by cervical dislocation and the brains processed.

### Tissue processing

Brains were dissected, immersion fixed and cryoprotected in 4% paraformaldehyde with 30% sucrose for up to 12 hours at 4°C. The brains were then embedded in gelatine and stored in a 30% sucrose solution at 4°C. Brains were sectioned into 40 μm slices on a vibratome (Leica VT1200S). Sections were collected in 10 parallel series and stored in tubes of 0.12 M phosphate-buffered saline (PBS) and 0.1% sodium azide at 4°C. For orientation, a hole was made with a needle in the left or right hemisphere of the brain (outside of the hypothalamus).

### Single-label immunohistochemistry (IHC)

IHC was done using a Vector labs Elite ABC kit (Brunschwig Chemie, Amsterdam) on free-floating sections. Unless otherwise stated, all steps were done at room temperature and by placing the sections on a low speed shaker to help evenly expose the sections to the solutions. Washing steps were in 0.05 M tris-buffered saline (TBS) for 5 minutes and repeated three times. Sections were placed into 0.12 M PBS for 10 minutes. Sections were then washed before incubation in 0.3% hydrogen peroxide and methanol for 10 minutes to remove endogenous peroxidase. After another washing step, the sections were incubated for 1 hour in 5% normal goat serum (NGS) blocking buffer in 0.03% triton-X100 in TBS (TBST). Excess buffer was removed and the slides incubated in primary antiserum for vasotocin (AVT) (rabbit anti-AVT 1:2500, kind gift from Prof. S. Blähser), or TH (rabbit anti-TH 1:2000, Chemicon AB152) in 1% NGS-TBST blocking buffer for 1 hour at room temperature and then at 4°C overnight. The following day, sections were washed twice in TBST before incubation for 1 hour in secondary antiserum (goat anti-rabbit IgG 1:200, DAKO, Denmark) in 1% NGS-TBST blocking buffer. After washing, sections were incubated in 1% avidin/biotin solution (Vectastain ABC-Elite kit) in TBS for 45 minutes. Sections were then washed before being stained with 3,3'-Diaminobenzidine (DAB, Sigma) with 0.02% H_2_O_2_ peroxidase activity in TBS (AVT: 60–80 seconds, TH: up to 150 seconds), then washed again. Finally, sections were mounted onto SuperFrostplus^®^ slides and processed through a clearing and dehydration series of ethanol and xylene to remove lipids and water, and cover-slipped using DePeX (Serva Electrophoresis, Heidelberg).

### Antisera specificity

To prevent cross-reaction to the related peptide mesotocin, the AVT specific rabbit serum was absorbed to oxytocin (the mammalian equivalent of mesotocin) coupled to CNBR-activated Sepahrose4B according to manufacturers protocol (GE Healthcare, the Netherlands) and the non-binding fraction of the antiserum was used. In addition, the specificity of the antiserum specific for AVT in chicken tissue was demonstrated by replacing the primary antibody with normal rabbit IgG as a control for non-specific interaction.

### Visualizing and analysis

One series was stained using anti-TH, and one series using anti-AVT. The distance between each consecutive section in the series was 400 μm. Division of the cells into subdivisions of the hypothalamus was based on a stereotaxic atlas of the chick brain (Puelles et al. [48]). Table 1 lists all areas and nuclei where cell counts were made. The average number of immunoreactive cells per area and per individual was divided by the number of sections containing that area and compared between treatment groups. The total number of immunoreactive cells per brain, without being divided by the number of sections containing the neuron type, was also compared between treatment groups. Stained sections were imaged on an Olympus BX51 microscope at magnification ×20 and ×40. An Olympus E330 digital camera was used with an Olympus BX40 microscope to take the images, which were processed using the Adobe Photoshop CS3 program. Counts of neurons were conducted live at magnification ×40. The observer was blind to the genetic line and rearing condition of each subject. All statistical analysis was calculated using SAS 9.2 (SAS Institute, Cary, NC) software. Because data was not normally distributed, non-parametric testing was employed. For comparison of hypothalamus neuron counts, Friedman's Two-way Nonparametric ANOVA with the factors Genetic Line and Rearing Conditions was used. Staining of all sections was spread across four batches. Possible batch artifacts were investigated and no significant effect was found, therefore staining batch was not considered as a factor for analysis. For the TH data, the LML HEN treatment group only had 6 brains due to fixation issues. Where appropriate, the four genetic line by rearing conditions groups were compared using Fisher’s least significant differences (LSD) post-hoc analysis. The null hypothesis was always that there is no difference between treatment groups. P values were considered significant at <0.05 unless otherwise stated.

### Correlation with behavioral data

To determine whether the histological data was related to behavior in the hens at a young age, correlations were determined between the histological data from the present study and previously published open field data. For the correlations, only the subset of hens included in the present histological study was included. The hens in the current experiment were exposed to an open field and observed at 6 weeks of age, as previously described [8]. Briefly, each focal bird was tested in an open-field test for 5 min. The open field consisted of a 1.25 × 1.25 m observation pen, which was divided into 5 × 5 squares by white markings, measuring 25 × 25 cm each. The front wall was made of Perspex, through which a camera recorded the area of the pen, allowing the observer to record the behavior using the software package “The Observer” (Noldus Information Technology BV, Wageningen, The Netherlands) from a video screen in an adjacent room. The latencies to vocalize, stand up and walk, as well as the number of distress calls and the number of steps were recorded using focal sampling. Birds were tested in a random order, alternating between the different housing pens. A single person conducted all tests and behavioral observations. Birds were tested between 0830 and 1630 h. Treatments were equally distributed over testing times.

To avoid a very large number of correlations, which would increase the likelihood of significant correlations by chance, only variables from the open field and histological measures were included in the correlation analysis that showed significant differences or trends in an ANOVA. Because a large number of variables were not normally distributed, Spearman Rank Correlations (SAS CORR SPEARMAN procedure) were calculated using SAS 9.2 (SAS Institute, Cary, NC) software. For the open field data, this meant the variables “latency to stand”, “latency to walk”, “number of distress calls”, and “number of steps”. We confirmed significant effects as described in Rodenburg et al. [8] in the subset of animals used in the current histological experiments for these variables (SAS GLM procedure; data not shown). The following histological data were included for correlation analysis: AVT positive neurons in MPA; AVT positive neurons in PaPC; AVT positive neurons in LPO; TH positive neurons in VMH; TH positive neurons in DM; TH positive neurons in Pe; and TH positive neurons in RM.

## Competing interests

The authors declare that they have no competing interests.

## Authors’ contributions

SEH conducted laboratory work, quantified sections, organized data, conducted statistics, and drafted the manuscript. ECZ sectioned hen brains and supervised laboratory work. FJCMvE aided in identifying hypothalamic areas in the hens, and with planning a quantification strategy for the histology. TBR provided the LML and CL hens, conducted the behavioral studies in the hens, aided in conceiving the histological study, and edited drafts of the manuscript. PJSvK conducted the laboratory work related to antibody specificity. FJvdS aided in conceiving the research question, conducted statistics, and edited drafts of the manuscript. REN conceived the histology study, designed the histology study, supervised laboratory work, and finalized the manuscript. All authors read and approved the final manuscript.

## References

[B1] Brunberg E Jensen P Isaksson A Keeling L Feather pecking behavior in laying hens: hypothalamic gene expression in birds performing and receiving pecks Poult Sci 2011 90 1145 1152 21597052 10.3382/ps.2010-00961

[B2] Harlander-Matauschek A Rodenburg TB Applying chemical stimuli on feathers to reduce feather pecking in laying hens Appl Anim Behav Sci 2011 132 146 151

[B3] Nordquist RE Heerkens JLT Rodenburg TB Boks S Ellen ED van der Staay FJ Laying hens selected for low mortality: behaviour in tests of fearfulness, anxiety and cognition Appl Anim Behav Sci 2011 131 110 122

[B4] Angevaare MJ Prins S van der Staay FJ Nordquist RE The effect of maternal care and infrared beak trimming on development, performance and behavior of Silver Nick hens Appl Anim Behav Sci 2012 140 70 84

[B5] Rodenburg TB van Krimpen MM de Jong IC de Haas EN Kops MS Riedstra BJ Nordquist RE Wagenaar JP Bestman M Nicol CJ The prevention and control of feather pecking in laying hens: identifying the underlying principles World's Poultry Science Journal 2013 69 361 374

[B6] Ellen ED Muir WM Teuscher F Bijma P Genetic improvement of traits affected by interactions among individuals: Sib selection schemes Genetics 2007 176 489 499 17409074 10.1534/genetics.106.069542 PMC1893021

[B7] Bolhuis JE Ellen ED Van Reenen CG De Groot J Ten Napel J Koopmanschap RE Reilingh GDV Uitdehaag KA Kemp B Rodenburg TB Effects of genetic group selection against mortality on behavior and peripheral serotonin in domestic laying hens with trimmed and intact beaks Physiol Behav 2009 97 470 475 19341749 10.1016/j.physbeh.2009.03.021

[B8] Rodenburg TB Uitdehaag KA Ellen ED Komen J The effects of selection on low mortality and brooding by a mother hen on open-field response, feather pecking and cannibalism in laying hens Anim Welfare 2009 18 427 432

[B9] Rodenburg TB Bolhuis JE Koopmanschap RE Ellen ED Decuypere E Maternal care and selection for low mortality affect post-stress corticosterone and peripheral serotonin in laying hens Physiol Behav 2009 98 519 523 19699216 10.1016/j.physbeh.2009.08.006

[B10] Broom DM Zanella AJ Brain measures which tell us about animal welfare Anim Welfare 2004 13 S41 S45

[B11] Nishi M Horii-Hayashi N Sasagawa T Matsunaga W Effects of early life stress on brain activity: Implications from maternal separation model in rodents Gen Comp Endocrinol 2013 181 306 309 23032077 10.1016/j.ygcen.2012.09.024

[B12] Barha CK Pawluski JL Galea LAM Maternal care affects male and female offspring working memory and stress reactivity Physiol Behav 2007 92 939 950 17716698 10.1016/j.physbeh.2007.06.022

[B13] Menard J Champagne D Meaney M Variations of maternal care differentially influence 'fear' reactivity and regional patterns of cFos immunoreactivity in response to the shock-probe burying test Neuroscience 2004 129 297 308 15501588 10.1016/j.neuroscience.2004.08.009

[B14] Francis D Meaney M Maternal care and the development of stress responses Curr Opin Neurobiol 1999 9 128 134 10072372 10.1016/s0959-4388(99)80016-6

[B15] Field SE Rickard NS Toukhsati SR Gibbs ME Maternal hen calls modulate memory formation in the day-old chick: the role of noradretialine Neurobiol Learn Mem 2007 88 321 330 17507256 10.1016/j.nlm.2007.04.001

[B16] Madec I Gabarrou J Pageat P Influence of a maternal odorant on copying strategies in chicks facing isolation and novelty during a standardized test Neuroendocrinol Lett 2008 29 507 511 18766150

[B17] de Margerie E Peris A Pittet F Houdelier C Lumineau S Richard-Yris M Effect of mothering on the spatial exploratory behavior of quail chicks Dev Psychobiol 2013 55 256 264 22362163 10.1002/dev.21019

[B18] Houdelier C Lumineau S Bertin A Guibert F De Margerie E Augery M Richard-Yris M Development of fearfulness in birds: Genetic factors modulate non-genetic maternal influences PLoS One 2011 6 e14604 21298038 10.1371/journal.pone.0014604 PMC3029269

[B19] Edgar JL Paul ES Nicol CJ Protective mother hens: cognitive influences on the avian maternal response Anim Behav 2013 86 223 229

[B20] Nordquist RE Zeinstra EC Rodenburg TB van der Staay FJ Effects of maternal care and selection for low mortality on tyrosine hydroxylase concentrations and cell soma size in hippocampus and nidopallium caudolaterale in adult laying hen J Anim Sci 2013 91 137 146 23048145 10.2527/jas.2012-5227

[B21] Kops MS de Haas EN Rodenburg TB Ellen ED Korte-Bouws GAH Olivier B Güntürkün O Korte SM Bolhuis JE Selection for low mortality in laying hens affects catecholamine levels in the arcopallium, a brain area involved in fear and motor regulation Behav Brain Res 2013 257 54 61 24076385 10.1016/j.bbr.2013.09.035

[B22] Frodl T O'Keane V How does the brain deal with cumulative stress? A review with focus on developmental stress, HPA axis function and hippocampal structure in humans Neurobiol Dis 2013 52 24 37 22426398 10.1016/j.nbd.2012.03.012

[B23] Xie J Kuenzel WJ Allen DL Jurkevich A Differential neural activation in the septo-hypothalamic region following sexual and agonistic behavior in male broiler breeders Poult Sci 2008 87 18

[B24] Kuenzel WJ Jurkevich A Molecular neuroendocrine events during stress in poultry Poult Sci 2010 89 832 840 20308419 10.3382/ps.2009-00376

[B25] Herman JP Flak J Jankord R Chronic stress plasticity in the hypothalamic paraventricular nucleus Advances in Vasopressin and Oxytocin: from Genes to Behaviour to Disease 2008 170 353 364 10.1016/S0079-6123(08)00429-9 PMC3641577 18655895

[B26] Adkins-Regan E Neuroendocrinology of social behavior ILAR J 2009 50 5 14 19106448 10.1093/ilar.50.1.5

[B27] Goodson JL Deconstructing sociality, social evolution and relevant nonapeptide functions Psychoneuroendocrinology 2013 38 465 478 23290368 10.1016/j.psyneuen.2012.12.005

[B28] Prasad B Sorg B Ulibarri C Kalivas P Sensitization to stress and psychostimulants - Involvement of dopamine transmission versus the HPA axis Ann NY Acad Sci 1995 771 617 625 8597435 10.1111/j.1749-6632.1995.tb44714.x

[B29] Belda X Armario A Dopamine D1 and D2 dopamine receptors regulate immobilization stress-induced activation of the hypothalamus-pituitary-adrenal axis Psychopharmacology (Berl) 2009 206 355 365 19621214 10.1007/s00213-009-1613-5

[B30] Kjaer JB Hjarvard BM Jensen KH Hansen-Moller J Larsen ON Effects of haloperidol, a dopamine D2 receptor antagonist, on feather pecking behaviour in laying hens Appl Anim Behav Sci 2004 86 77 91

[B31] Goodson JL Saldanha CJ Hahn TP Soma KK Recent advances in behavioral neuroendocrinology: insights from studies on birds Horm Behav 2005 48 461 473 15896792 10.1016/j.yhbeh.2005.04.005 PMC2570788

[B32] O'Connell LA Hofmann HA The Vertebrate mesolimbic reward system and social behavior network: a comparative synthesis J Comp Neurol 2011 519 3599 3639 21800319 10.1002/cne.22735

[B33] Moons L Vangils J Ghijsels E Vandesande F Immunocytochemical localization of L-Dopa and dopamine in the brain of the chicken (Gallus-Domesticus) J Comp Neurol 1994 346 97 118 7962714 10.1002/cne.903460107

[B34] Sanchez F Panzica G Vigiettipanzica C Aste N Carretero J Vazquez R A comparative-analysis of the vasotocin and vasopressin systems in the chicken and rat hypothalamus - an immunocytochemical study J Hirnforsch 1991 32 27 37 1811016

[B35] Swanson L Hartman B Central adrenergic system - Immunofluorescence study of location of cell bodies and their efferent connections in rat utilizing dopamine-beta-hydroxylase as a marker J Comp Neurol 1975 163 467 505 1100685 10.1002/cne.901630406

[B36] Dudas B Baker M Rotoli G Grignol G Bohn MC Merchenthaler I Distribution and morphology of the catecholaminergic neural elements in the human hypothalamus Neuroscience 2010 171 187 195 20801195 10.1016/j.neuroscience.2010.08.050

[B37] Ruggiero DA Ross CA Anwar M Park DH Joh TH Reis DJ Distribution of neurons containing phenylethanolamine N-methyltransferase in medulla and hypothalamus of rat J Comp Neurol 1985 239 127 154 2864362 10.1002/cne.902390202

[B38] Thind K Goldsmith P Corticotropin-Releasing Factor neurons innervate dopamine neurons in the periventricular hypothalamus of juvenile macaques - Synaptic evidence for a possible companion neurotransmitter Neuroendocrinology 1989 50 351 358 2571955 10.1159/000125249

[B39] Crosby E Woodburne R Neuroanatomy Prog Neurol Psychiatry 1955 10 1 15 13266864

[B40] Rodenburg TB van Oers K Interactions between behaviour and genetics in wild and domestic bird populations9th World Congress on Genetics Applied to Livestock Production 2010 99

[B41] Wood R Newman S Androgen receptor immunoreactivity in the male and female Syrian hamster brain J Neurobiol 1999 39 359 370 10363909 10.1002/(sici)1097-4695(19990605)39:3<359::aid-neu3>3.0.co;2-w

[B42] Clarkson J Herbison AE Dual phenotype kisspeptin-dopamine neurones of the rostral periventricular area of the third ventricle project to gonadotrophin-releasing hormone neurones J Neuroendocrinol 2011 23 293 301 21219482 10.1111/j.1365-2826.2011.02107.x

[B43] Sartsoongnoen N Kosonsiriluk S Prakobsaeng N Songserm T Rozenboim I El Halawani M Chaiseha Y The dopaminergic system in the brain of the native Thai chicken, Gallus domesticus: localization and differential expression across the reproductive cycle Gen Comp Endocrinol 2008 159 107 115 18765240 10.1016/j.ygcen.2008.08.002

[B44] Prakobsaeng N Sartsoongnoen N Kosonsiriluk S Chaiyachet O Chokchaloemwong D Rozenboim I El Halawani M Porter TE Chaiseha Y Changes in vasoactive intestinal peptide and tyrosine hydroxylase immunoreactivity in the brain of nest-deprived native Thai hen Gen Comp Endocrinol 2011 171 189 196 21266179 10.1016/j.ygcen.2011.01.007

[B45] Numan M Numan M Projection sites of medial preoptic area and ventral bed nucleus of the stria terminalis neurons that express Fos during maternal behavior in female rats J Neuroendocrinol 1997 9 369 384 9181491 10.1046/j.1365-2826.1997.t01-1-00597.x

[B46] Sharp PJ Broodiness and broody control Poult Sci Symp Ser 2009 29 181 205

[B47] Melo AI Hernandez-Curiel M Hoffman KL Maternal and peer contact during the postnatal period participate in the normal development of maternal aggression, maternal behavior, and the behavioral response to novelty Behav Brain Res 2009 201 14 21 19428611 10.1016/j.bbr.2009.01.023

[B48] Puelles L Martinez-de-la-Torre M Paxinos G Watson C Martinez S The Chick Brain in Stereotaxic Coordinates 2007 San Diego: Elsevier Academic Press

